# Supplementary material for: Evolution of Angiogenic Factors in Pregnant Patients with Breast Cancer Treated with Chemotherapy
Source: Cancers (Basel). 2021 Feb 23;13(4):923. doi: 10.3390/cancers13040923 (PMC7926500; doi:10.3390/cancers13040923)
Supplement: Supplementary file 1 [file cancers-13-00923-s001.pdf]

Table S1. Baseline characteristics of 12 patients with breast cancer diagnosed during pregnancy.

Variable	No. (%)
Age, years, mean (range)	37 (30-43)
Trimester of gestation	
First	5 (42)
Second	5 (42)
Third	2 (16)
Mammography, BI-RADS category	
0	1 (8)
1	2 (17)
2	0
3	0
4	3 (25)
5	1 (8)
6	5 (42)
Ultrasound	
Suspicious findings	12 (100)
Clinical staging	
0	0
I	0
IA	3 (25)
IB	1 (8)
IIA	4 (34)
IIB	3 (25)
III	0
IV	1 (8)
Histological grade	
1	0
2	3 (25)
3	9 (75)
Immunohistochemistry	
Positive hormone receptors*	5 (42)
HER2 overexpression¥	5 (42)
Triple negative	4 (34)
Histological type	
Infiltrating Ductal Carcinoma	11 (92)
Infiltrating Lobal Carcinoma	1 (8)

* Estrogen receptor and/or progesterone receptor score >1%

¥ HercepTest positive (+++) or (++) and in situ hybridization (FISH or ISH) positive for HER2 following ASCO-CAP guidelines 2013.

Table S2. Complications during gestation in 10 patients with breast cancer diagnosed during pregnancy. Two patients do not have any complication.

Case#	Complication	GA	Treatment/outcome	Relation with treatment	Comment
2	Fever	20	Paracetamol.	Possible	
	Constipation	16	Laxant.	Possible	
		23	Laxant (high dose).	Possible	
	Abnormal intrapartum fetal heart rate tracing	37	Intrapartum C-Section.	Possible	False positive. Normal postnatal pH.
3	Mucositis	25	Viscous lidocaine.	Yes	
	Asthenia	26	Non-specific treatment.	Possible	
	Anaemia	28	Intravenous iron.	Possible	
	Neutropenia	32	Chemotherapy discontinuation.	Yes	
	Preterm threatened labor	32	Admission. Tocolytic treatment administration and fetal lung maturation.	Possible	Chemotherapy-induced stress.
	Preterm rupture of membranes	35	Admission and tocolytic treatment. Anticipation of termination of pregnancy.	Possible	Chemotherapy-induced stress.
4	Mucositis	36	Viscous lidocaine.	Yes	
	Suspicion of fetal anaemia	36	Close monitoring.	Possible	False positive. Suspicion of fetal anaemia (MCA>95) was discarded. after delivery.
5	Asthenia	16	Non-specific treatment.	Possible	
	Constipation	19	Laxant.	Possible	
	Itching	31	Polaramine.	Possible	
	Preterm threatened labour	33	Admission. Tocolytic treatment administration and fetal lung maturation. Anticipation of termination of pregnancy.	Possible	Chemotherapy-induced stress.
	Preterm rupture of membranes				
6	Cervical shortening	30	Admission. Fetal lung maturation and fetal neuroprotection	Possible	Chemotherapy-induced stress.
	Nauseas	32	Antiemetic treatment.	Possible	
	Anaemia	32	Intravenous iron.	Possible	
7	Nauseas	5	Antiemetic treatment.	No	Clinical manifestation prior to chemotherapy.
	Vomit	18	Antiemetic treatment.	Possible	Secondary to chemotherapy. Appeared after the first administration of AC.
	Xeroderma	30	Skin hidratation.	Possible	
	Pruritus / Itching	30	Antihistaminic treatment,	Possible	
8	Constipation	30	Laxant.	Possible	
	Gastritis	30	Ranitidina.	Possible	
10	Nauseas	23	Antiemetic treatment.	Possible	
	Constipation	32	Laxant.	Possible	
	Heartburn	30	Ranitidine.	Possible	
	Gestational diabetes	33	Insulinotherapy.	No	Previous gestational diabetes antecedent
	Asthenia	33	Admission. Patient rest.	Possible	

	Suspicion of fetal anaemia	35	Close monitoring	Possible	False positive. Suspicion of fetal anaemia (MCA>95) was discarded after delivery.
11	Asthenia	27	Non-specific treatment.	Possible	
	Xerostomia (Dry mouth)	27	Non-specific treatment.	Possible	
12	Anaemia	23, 28	Transfusion and iron administration.	Possible	
	Asthenia	23	Non-specific treatment.	Possible	
	Suspicion of fetal anaemia	30	Admission. Close monitoring.	Possible	False positive. Suspicion of fetal anaemia (MCA>90) was discarded after delivery.
